# Effectiveness of an intervention for managing victimization risks related to societal participation for persons with severe mental illness: a cluster RCT study protocol

**DOI:** 10.1186/s12888-018-1831-7

**Published:** 2018-08-02

**Authors:** Wendy M. M. Albers, Diana P. K. Roeg, Yolanda Nijssen, Inge M. B. Bongers, Jaap van Weeghel

**Affiliations:** 1 Tranzo Scientific Centre for Care and Welfare, Department of Social and Behavioural Sciences, Tilburg University, PO Box 90153, 5000 Tilburg, LE Netherlands; 2 GGzE Centre for Mental Health Care, PO BOX 909, 5600 Eindhoven, AX Netherlands; 3 Phrenos Centre of Expertise, PO Box 1203, 3500 Utrecht, BE Netherlands; 4 Dijk en Duin Mental Health Centre, Parnassia Group, PO Box 305, 1900 Castricum, AH Netherlands; 5 Erasmus Center for Health Care Governance, Erasmus University, PO Box 1738, 3000 Rotterdam, DR Netherlands

**Keywords:** Victimization, Rehabilitation, Recovery, Societal participation, Severe mental illness, Stigma, Risk management

## Abstract

**Background:**

People with severe mental illness (SMI) are more likely to experience criminal victimization than other community members. In addition, (self-) stigma and perceived discrimination are highly prevalent in this group. These adversities in the social context often have major adverse effects on the rehabilitation and recovery of these persons. Current practice, however, lacks instruments to address these issues. As a reaction, the Victoria intervention was developed and pilot-tested with client representatives, professionals, trainers and researchers. The Victoria intervention is a method for community mental health care workers to expand their awareness of this topic and support them in assessing victimization and incorporate appropriate services, including trauma screening and rehabilitation services, in their health care planning. For clients, the Victoria intervention aims to increase their awareness, active management of possible victimization risks and promote safe social participation. As a new intervention, little is known about its use in real practice and its effects on client outcomes.

**Methods/design:**

To determine the feasibility and effectiveness of this intervention, a process evaluation and a first cluster randomized controlled trial (RCT) will be carried out. Outpatients from eight Flexible Assertive Community Treatment (F-ACT) teams from two mental health care (MHC) organizations in the Netherlands are included in the study. Teams in the intervention group will receive three half-day training sessions, and bi-monthly supervision meetings for 18 months. Teams in the control group provide care as usual. For the process evaluation, a multi-method design is used. To assess effects on client outcomes, clients will be interviewed about their experiences on victimization and societal participation using validated questionnaires at baseline, and after 9 and 18 months.

**Discussion:**

This study is the first to evaluate an intervention aiming at recognition of victimization, (self-) stigma and perceived discrimination, and targeting outpatients’ insights into possible risks and coping skills to tackle these risks to enhance safe societal participation. Results of this study may validate the Victoria intervention as a practice to better manage risk for adversities related to societal participation.

**Trial registration:**

Dutch Trial Register (NTR): 5585, date of registration: 11–01-2016.

## Background

In most western countries, deinstitutionalization has led to an increase of community-based care with a focus on promoting the recovery and societal integration of people with severe mental illness (SMI) [1]. To achieve successful integration into community life, psychiatric rehabilitation methods were developed in order to support people with SMI to regain a meaningful life and valued social roles [2, 3]. Within the field of psychiatric rehabilitation, several evidence-based practices can be identified. The Boston Approach to Psychiatric Rehabilitation (BPR) has proven to be effective in promoting new perspectives, role functioning, and life satisfaction [4, 5]. Another evidence-based rehabilitation intervention is the Individual Placement and Support (IPS) model of supported employment [6]. Employment specialists successfully support clients to search for and get a job, and also coach clients about working situations in order to maintain employment.

There are still substantial challenges to work on in community-based care, especially concerning supporting clients in their social and community participation. A lack of support in these areas may lead to unemployment, poverty, social isolation and even imprisonment [7]. Studies have also shown that these negative consequences may lead to an increase of victimization rates [8–10]. Not only are people with SMI more likely to experience victimization than other community members, in contrast to popular public belief, they are also more likely to become a victim of a crime rather than being the perpetrator [11–15]. Prevalence rates of violent victimization among persons with SMI range between 7 and 56% in the previous year – 11 times greater than the general population [8]. A recent nationwide study in the Netherlands examined prevalence rates of several types of victimization and found that almost 20% of people with SMI were a victim of serious crimes in the previous year, such as sexual harassment/assault, violence, and physical assault [11]. In most cases, the victim knew the perpetrators of these crimes.

Likewise, internalized stigma and perceived discrimination are highly prevalent in people with SMI [16, 17]. Brohan et al. [17] found that more than 40% of people with schizophrenia or other psychotic disorders reported internalized stigma and almost 70% perceived discrimination. Self-stigma entails becoming aware of the negative stereotyping of people with mental illness and eventually applying it to one’s self. This may result in lower self-esteem and self-efficacy, and eventually leads to avoiding behavior that interferes with achieving life goals and social integration – the so-called ‘why try’ effect [18]. High levels of self-stigma and perceived discrimination were also associated with a lower number of social contacts, and difficulties finding employment [17, 19, 20]. Many experience this stigma and discrimination on a regular basis in their daily and social activities.

Adversities such as victimization, stigmatization and discrimination, whether experienced or anticipated, are important barriers for personal recovery and social participation [14]. Anticipating stigmatization or discrimination, people with SMI tend to refrain from social interaction or daily activities to prevent future rejection or victimization [16]. This can lead to social deprivation, loneliness, and consequently to a loss of confidence, lower self-efficacy and lower quality of life [16, 17, 21]. For this reason, in this study victimization is related to all adversities people with SMI may experience in their social and community functioning, including stigmatization, discrimination and criminal victimization, e.g. robbery, sexual assault, and property crimes.

Flexible Assertive Community Treatment (F-ACT) is a predominant type of community mental health care (MHC) for people with SMI in the Netherlands. F-ACT teams are meant to support clients with rehabilitation goals and wishes to regain valuable roles in community [22]. Nevertheless, these teams experience difficulties in thoroughly supporting clients in employment, social support and social functioning [21]. In practice, the focus and availability of these teams tend for several reasons to be more on diagnosis, treatment, and crisis management, rather than on rehabilitation and community support [23].

Another reason for the lack of attention towards societal participation, and the impact of adversities on this, is that many mental health professionals (MHPs) fear an increase of symptoms or relapse by addressing adversities related to societal participation [24]. Despite the high prevalence and major effects of victimization, discrimination and stigmatization, it is not self-evident that MHPs address victimization or other adversities, at least not in a systematic way, both at intake and throughout their treatment [21, 25]. However, current studies suggest that talking about victimization and other adversities does not lead to an increase of symptoms or relapse. On the contrary, it was shown that talking about it leads to more acknowledgement for and understanding of the situation, for both the MHP and the client [26–28]. Moreover, it provides tools to prevent or to cope with possible risks when engaging in future social situations.

Social recovery is inevitably associated with ups and downs when it comes to regaining valued roles in society. This is recognized by advocates of the consumer advocates, who developed the concept of ‘dignity of risk’ [29, 30]. Dignity of risk emphasizes personal choice and self-determination, which are also two central concepts in social recovery [31], and assumes that people with SMI have the right to self-manage decisions about wellness, employment, and social contacts, and profit more from taking risks than from avoiding them. Taking into account the high rates of victimization in this group, risks taken on their road to recovery, however, should be well assessed and managed. This positive risk management perspective has also been promoted by the UK department of Health [32]. On the one hand, it is necessary for both clients and professionals to make realistic assessments of the clients’ abilities regarding societal participation; on the other hand, clients need to acquire skills to minimize adversities [24].

The Victoria intervention for community MHC workers is the first to incorporate this positive risk approach in a psychiatric rehabilitation method. Although there is growing recognition for the benefits of exposure and other trauma-focused treatments for persons with SMI, including persons suffering from psychosis [33], prevention of victimization in relation to social participation is not addressed yet. Likewise, in the field of recovery-oriented care there is increasing evidence that addressing (risks on) victimization or other adversities may also be beneficial for clients on their road to recovery [26–28]. However, in community MHC, practitioners do not adopt this positive risk approach yet, and do not address adversities in the social context in a structural or systematic way [34].

The Victoria intervention is a method for community MHC workers to increase their awareness about the topic and support them in assessing victimization and incorporate appropriate services, including trauma screening and rehabilitation services, in their health care planning. For clients, the Victoria intervention aims to increase their awareness, active management of possible victimization risks and promotes safe social participation [26, 35].

### Objective and research questions

The Victoria intervention is a novel intervention. Although the intervention is thoughtfully designed and piloted, due to its novelty it is important to study the implementation process and its context. Therefore, we will perform both a trial and a process evaluation to study the effectiveness and feasibility of this intervention. In addition, it is not only important to examine whether the intervention is effective as a whole, it is also relevant to further examine which clients will or will not benefit from the intervention [36].

The aim of this initial trial on the Victoria intervention is to gain insight into the implementation process and effectiveness of the Victoria intervention, on both the team level and the client level, in reducing victimization, including other adversities, and increasing societal participation. The primary research questions are:

1. To what extent is the Victoria intervention implemented as intended and how is this new intervention perceived by the MHP?
2. Does applying the Victoria intervention lead to increased societal participation and decreased victimization compared to care as usual (CAU) for clients of outpatient teams with SMI?

Secondary research questions are:

1. Is the Victoria intervention an effective intervention for clients with SMI with regard to acknowledgement of adversities, quality of life, psychosocial functioning, and self-efficacy, compared to CAU?
2. Is the Victoria intervention an effective intervention compared to CAU, with regard to awareness and acknowledgement of victimization by MHPs, and their insight in societal participation of the client?

## Methods

### Design

This study includes: 1) a process evaluation and 2) a two-armed multi center cluster randomized controlled trial (RCT) to follow the implementation process and determine the effectiveness of the Victoria intervention. Participants include adult clients of eight F-ACT teams who are interviewed about their victimization experiences and rehabilitation process at three points in time: at baseline, and after 9 and 18 months. Study characteristics are described according to SPIRIT guidelines [37]. This study was approved by the Medical Ethical Committee of the Elisabeth Hospital in Tilburg (NL53845.028.15) on the 18th of November 2015 for all participating sites. The study is registered with the Dutch Trial Register (NTR 5585).

### Setting and team structure

The Victoria study will be performed in F-ACT teams in two MHC organizations, one in the north-west and one in the south of the Netherlands. F-ACT is a flexible model of assertive community treatment (ACT), in which it is possible to switch from intensive treatment (ACT) or crisis management at the one hand to individual case management and multidisciplinary treatment on the other, and is considered the predominant type of community MHC for persons with SMI in the Netherlands [38]. F-ACT teams are, like ACT teams, multi-disciplinary, and consist of a variety of MHPs including a psychiatrist, psychiatric nurses, and an expert-by-experience. One of the benefits of F-ACT is that clients receive care within one team and can build a relationship; this continuity of care creates better opportunities for recovery and rehabilitation [38]. The eight participating teams have a total caseload of over 1500 clients.

### Clients

The client population consists of people with SMI, receiving outpatient care in one of the participating F-ACT teams. In the Netherlands, approximately 281,000 people have SMI, of whom 160,000 actually receive MHC [39]. Although these people have common problems and needs, this is a heterogeneous group with a range of psychiatric disorders. People with psychotic disorders form the largest group (60%); other prevalent diagnoses are bipolar disorder, anxiety disorder, personality disorder, and drug- or alcohol addiction, often in combination with each other. From the participating teams, all clients will be asked to participate in the study, following an informed consent procedure.

**Fig. 1:**
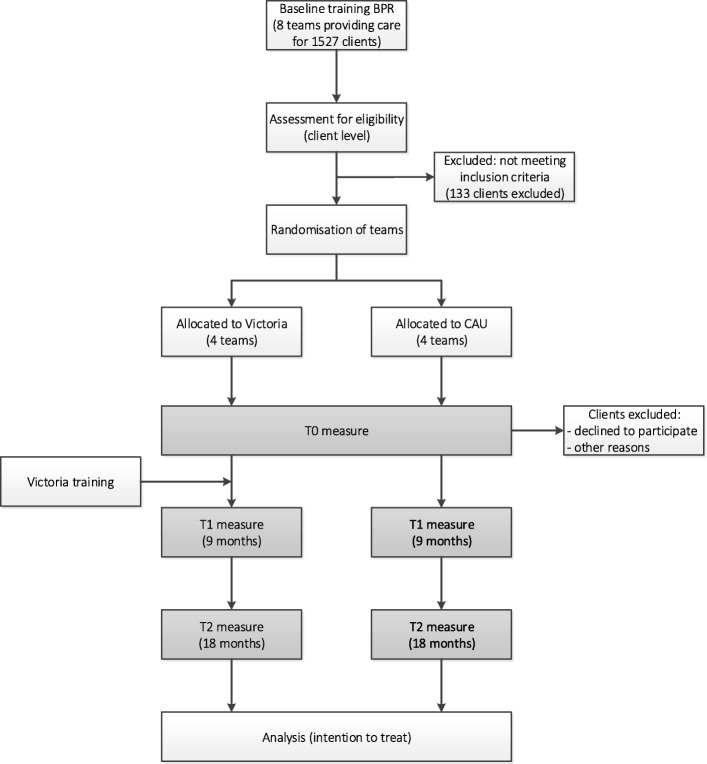
Flow chart of the study design. Note: ‘Other reasons’ are for example: prolonged admission, death, or imprisonment

Clients are eligible for participation if they receive care from the participating teams at the moment of recruiting. Clients will be excluded from the study if they, according to their case manager, meet one of the following criteria: younger than 18 years old, not having sufficient understanding of the Dutch language, not being capable of completing the interview due to cognitive impairment, having severe symptomatology, or psycho-organic disorder, and being admitted to a psychiatric hospital, or staying in prison during the recruitment period (Fig. 1).

### Victoria intervention

The Victoria intervention is developed together with ‘Stichting Rehabilitatie ‘92’, and consists of four steps (Exploring, Analyzing, Clarifying context, Future steps) in discussing victimization and other adversities related to societal participation with the client. The MHP who have received training in this module are able to use this intervention for clients that are facing difficulties with societal participation. The goal of these conversations is acknowledgement of these adversities and their impact on daily life by the MHP, and for the client to become more aware of risky situations. In these conversations, the client is given tools and supported to adequately cope in future risky situations. This asks for attitude changes in MHPs during training with the ultimate aim to expand societal participation and decrease victimization for the client. The Victoria intervention can be regarded a preamble intervention, to be used as part of the starting phase in rehabilitation methods, such as the BPR. Additionally, when a client experiences difficulties in one of the rehabilitation phases, and stagnates in societal participation, it can be used as a restart intervention.

Within the conversation context of Victoria, the MHP creates room for the client to tell his or her own story including experiences with victimization and other adversities, and supports the client in rediscovering his/her goals and wishes for societal participation. By following the four steps, Victoria supports the MHC to shift focus from, which is currently the case in FACT teams, crisis management to more recovery-oriented care.

The first step of the Victoria intervention is *Exploring* and involves checking the level of activity in the following domains: housing, social contacts, education, and work. The MHP assesses whether the client is avoiding activities, or if progress on these domains is stagnating. If this is the case, together they examine whether this avoidance or stagnation is linked to experiences with victimization or other adversities related to societal participation.

The second step is *Analyzing* and involves investigation of this negative experiences together (who, what, where, when). It is crucial that the MHP uses active listening techniques while the client is elaborating on these adverse events. Furthermore, the professional tries to understand the intensity and related feelings of the specific experiences, but also tries to uncover the causes for the avoidance or stagnation. The overall goal in this step of the intervention is to get a full picture of the negative events, to recognize and acknowledge feelings related to these events, and to understand what caused the client to stop or avoid societal participation.

The third step entails *Clarifying the context* of the adverse events. The MHP examines together with the client what desire or wish undergirded the events, i.e. what was the client’s motive to engage in this activity. Again, it is critical that the MHP actively creates room to let the client tell his or her story. If the underlying desire or wish is clear, the client explains to the MHP how he/she would have wanted the situation to go, and what he/she hoped to achieve by engaging in this activity. This is the link to further rehabilitation services.

The fourth and final step of the intervention comprises *Discussing future steps.* Overall, there are three possible outcomes of the Victoria intervention. Firstly, another appointment may be necessary to further discuss the adverse events. Secondly, if the client feels the MHP acknowledges the intensity of these adverse events and corresponding feelings, the next step may be to start a rehabilitation action plan to work on the original rehabilitation goal, using rehabilitation services. Finally, if the victimization experience was intense, the MHP should investigate whether trauma treatment is needed. If there are indications for trauma, the 10-item Trauma Screening Questionnaire (TSQ) is used [40]. If 6 or more items are answered positively, trauma treatment with the F-ACT psychologist is advised.

#### Training professionals to use the Victoria intervention

In this study, the intervention teams receive will three half-day training sessions. Two trainers from ‘Stichting Rehabilitatie ‘92’ and one expert-by-experience facilitate the training sessions. These sessions will focus on explaining the background of the intervention (including theory) and explaining the four steps of the intervention. Additionally, the second and third training sessions will include practicing in small groups and plenary role-play. Preferably, real life cases of the teams are used and discussed. If these cases are too complex to use in role-play, several fictitious cases are available in hand outs. All MHPs receive the Victoria handbook, as well as a shorter hand out in the form of a factsheet, and the case examples. For the professionals to be able to use the Victoria intervention, no specific materials are necessary. In order to ensure that MHPs will bring the intervention into practice, supervision meetings will be offered every 6–8 weeks, during 18 months. In these meetings difficulties in practice based on real life cases are discussed. One of the Victoria trainers leads these meetings. In order to ensure that training sessions across the country will be similar, the content of the training was prepared together with the trainers, and all training sessions will be recorded and spread among other trainers. Finally, a short educational film will be shown in one of the supervision meetings, containing a good example of a Victoria conversation between a real rehabilitation coach and an expert-by-experience. The goal of this film is both improving practice and enlarging comparability of the training and supervision meetings across the country.

Part of the training and supervision meetings will be brainstorming about incorporating the Victoria intervention in day-to-day work. Incorporating Victoria in daily routines may include a short report of a conversation between the MHP and the client, but may also include discussion during a team meeting.

#### Care as usual

Professionals in the control group will continue to work according to CAU in F-ACT teams, including F-ACT practice and rehabilitation according to the BPR, which is the common rehabilitation approach in both participating organizations. The goal of the BPR is: “to assure that the person with a psychiatric disability possesses those physical, emotional, and intellectual skills needed to live, learn, and work in his or her own particular environment” [2]. The BPR supports clients in formulating their rehabilitation goals and wishes, in how to choose, get, and keep a preferred and valued role on several domains such as housing, social contacts, education, and work [2, 41].

To ensure that both sites are comparable on their knowledge and skills regarding the BPR, and to avoid differences in effect due to differences in rehabilitation skills of the team members, all participating teams receive the basic training in the BPR by the official Dutch BPR training center ‘Stichting Rehabilitation ‘92′, prior to randomization. This entails a 7-day training for all case managers, experts-by-experience, and occupational workers, where they will be taught theoretical principles and acquire practical skills. After training, professionals also receive supervision for 6 months where individual cases are discussed. After those 6 months, teams in the control group will continue to work according to principles of F-ACT and the BPR for the total duration of the trial. The BPR training and supervision is conducted prior to training and supervision of the Victoria intervention.

### Process evaluation

Because the Victoria is a novel intervention, in this first full cluster RCT, a process evaluation is highly relevant. A process evaluation is even more important in so-called ‘complex interventions’ [42, 43]. Complex interventions ask for a change of perspective in the MHP, but also operate in an organizational context that is difficult to influence or rule out [43]. When an RCT answers the question of whether the intervention works (or not), a process evaluation is performed to *understand* the possible effects of the intervention [42]. Therefore, a process evaluation is an enhancement of the RCT. In this process evaluation, the implementation and use of the Victoria intervention is evaluated on the following aspects: fidelity, feasibility, relevance and acceptation. More specifically, the focus will be on understanding the experiences of the clients who receive the intervention, but even more so on the professionals’ experiences with and perceptions of the intervention.

To achieve this, a multi-method design will be used. First, qualitative interviews will be carried out with several professionals and clients. MHPs of the outpatient teams are purposefully selected, including the trainers of the intervention and management staff from the two involved sites. Twenty clients participating in the RCT will be selected and asked for consent. These interviews will take place from a year after the training for the RCT. A topic list will be used to steer the interview. The interviews with the professionals will focus on understanding perceptions regarding the relevance of: the intervention, the training and the supervision meetings, the implementation as a whole, and the feasibility of using the intervention in real life. The clients will be asked if they noticed obvious differences in the conversations with their case managers with regard to adversities in the social context, and how they perceive these differences. Data will be audio recorded, transcribed verbatim, and coded using the ATLAS software program. Second, the supervision sessions will be recorded and analyzed to examine whether professionals implemented the Victoria intervention as intended, and to get a better picture of their experiences with and perceptions of the intervention. Third, during the supervision sessions, the professionals will be asked to fill out a checklist to inquire their knowledge about the steps of the intervention. This is done to measure fidelity of the intervention. Fourth, in the RCT questionnaires for the intervention teams, questions are added about the extent to which the professional has executed the intervention in real practice, and on the insight the professional has in potential adversities that act as a barrier for clients’ participation. Both the checklist and the questionnaire are analyzed using SPSS (version 22).

### Cluster RCT

#### Recruitment and consent

Clients eligible for participation receive a letter and brochure with information about the study, as approved by the ethical committee. In this letter, the themes of the study, questionnaire and time frame are explained. Clients are also informed that during the study they can withdraw at any time. After a two-week consideration period, the researchers will contact the client to ask if more information is needed and if they are willing to participate. If the client is willing to participate, a date, time, and place for the interview is scheduled. Clients will be asked to give their written informed consent before the start of the baseline interview. Participants will receive a compensation of 5, 10, and 15 euros for T0, T1, and T2 respectively.

Because randomization is performed at a team level and the Victoria intervention is considered a team approach, no separate informed consent is needed for the group randomization and consequently having the Victoria conversation. However, if the client wishes not to talk about societal participation and related adverse events, MHPs have to respect that.

#### Randomization and blinding

The participating teams will be randomly allocated to either the experimental or control condition by an independent senior researcher at Tilburg University, stratified by mental health organization. Cluster randomization was chosen as individual randomization would mean reassigning clients from their regular case managers and was therefore considered to be ethically undesirable. Moreover, cluster randomization reduces risks of contamination between the intervention and control group, as the intervention method is team based. We will monitor staff changes as well as clients switching teams to correct for in statistical analyses.

Due to the nature of the intervention, both the MHP and researchers cannot be unaware of the allocation to the conditions, but they are strongly advised not to disclose to the participating clients whether they receive care from an experimental or control team.

#### Measures

Table 1 provides an overview of the measurement instruments used. These instruments were chosen according to their comparability in national and international mental health research, and their psychometric characteristics. Duration and sensitivity to measure change were also taken into consideration. The first author as well as trained interviewers will carry out the interviews, which will take place on a location preferred by the client, at home or at team location. The interviewers were trained by explaining the topic list and using role-play, in order minimize bias due to inter-reviewer differences. Moreover, the first one or two interviews will be performed in dyads, with the researcher. Data will be entered into a secured database by researchers or research assistants. Participants’ names will be changed into randomly assigned numbers of which only two of the authors have the key.

**Table 1:** Overview of measurement instruments

Concept	Instrument	Level	T0	T1	T2^a^
Primary outcome measures					
Societal participation	Birchwood Social Functioning Scale (SFS)	Client	x	x	x
Criminal victimization	The Safety Monitor, section 4	Client	x	x	x
Discrimination and stigmatization	Discrimination and Stigma Scale (DISC-12)	Client	x	x	x
Perceived safety	The Safety Monitor, section 3	Client	x	x	x
Secondary outcome measures					
Acknowledgement of adversities	Structured questionnaire on feelings when discussing adversities	Client	x	x	x
Knowledge on rehabilitation and adversities	Structured questionnaire	MHP	x	x	x
Self-efficacy	Mental Health Confidence Scale (MHCS)	Client	x	x	x
Quality of Life	Manchester Short Assessment of Quality of Life (MANSA)	Client	x	x	x
General psycho-social functioning	The Health of the Nation Outcome Scales (HoNOS)	MHP	x	x	x
Additional and control measures					
Socio-demographic characteristics	Structured questionnaire	Client	x	x	x
Primary diagnosis	Structured questionnaire	MHP	x		
Number of years in MHC	Structured questionnaire	Client	x		
Social Support	Inventory of Social Reliance	Client	x	x	x
Neighborhood nuisance	The Safety Monitor, section 1 and 2	Client	x	x	x
Perpetration	The Safety Monitor, section 5	Client	x	x	x
FACT fidelity	CCAF scores	Team	x		x
Adherence to rehabilitation principles	Treatment plan (sample of 15% per team)	Client	x		x

^a^*T0* baseline, *T1* 9 months follow-up, *T2* 18 months follow-up

#### Primary outcome measures cluster RCT

1. The first primary outcome measure is *social participation *measured with the Birchwood Social Functioning Scale (SFS) [44]. It measures social functioning on seven domains: social engagement/withdrawal, interpersonal behavior, pro-social activities, recreation, independence-competence, independence-performance, and employment/occupation. The SFS is a reliable, valid, sensitive to change, instrument with a high internal consistency (α = 0.80) [44].

The second primary outcome measure is victimization related to societal participation and includes the following:

2. *Criminal victimization* will be measured with the Dutch version of the Safety Monitor, developed by the Dutch Ministry of Security and Justice [45], and strongly resembles the International Crime Victimization Survey [46]. It is a self-report questionnaire in which section 4 measures victimization on 15 crimes: burglary, theft from car, car theft, theft of other motorized vehicles, bicycle theft, (attempt to) robbery, theft (other than previously categorized), sexual intimidation or assault, threats (of violence), physical assault, vandalism, identity fraud, fraud with buying/selling items/services, hacking, cyber bullying. For each incident reported in the past 12 months, participants are asked to give more information about the incident.
3. *Perceived safety* will also be measured with the Safety Monitor. The participant is asked whether they ever feel unsafe (yes/no) and how often (often/sometimes/rarely).
4. *Discrimination and stigmatization* is assessed by the Discrimination and Stigmatization Scale (DISC-12) [47]. This scale consists of four subscales: unfair treatment, stopping self, overcoming stigma, positive treatment. The DISC-12 contains 32 items answered on a 4-point scale ranging from ‘no difference (0)’ to ‘a lot (3)’. A ‘not applicable’ answer is available when the participant was not involved in the described situation. Psychometric properties are considered good, Cronbach’s alpha is 0.78 and the inter-rater reliability ranges from 0.62 to 0.95.

#### Secondary outcome measures

The following secondary outcome measures will be used to gain more insight into the effects of the Victoria intervention.

1. *Acknowledgement of adversities* related to societal participation is assessed through a self-report questionnaire developed for this study.
2. *Knowledge on rehabilitation and adversities* will be measured through questions for MHP on the domains and phases of the BPR, and through questions on recent conversations about adversities. These questions are also developed for this study.
3. *Self-efficacy* in mental health-related beliefs is measured through the Mental Health Confidence Scale (MHCS) [48], with a 6-point Likert scale, ranging from ‘totally no confidence’ to ‘full confidence’. Cronbach’s alpha for the total scale is 0.93 [49].
4. To measure the *quality of life* the Manchester Short Assessment of Quality of Life (MANSA) is used [50, 51]. The MANSA has good internal consistency (α = 0.72) and is highly correlated (*r* = > 0.83 for each domain) with the Lancashire Quality of Life Profile (LQLP) [50]. The scale consists of 12 questions with a 7-point Likert scale ranging from ‘couldn’t be worse’ to couldn’t be better’ and 4 questions that are answered with yes/no.
5. *General psycho-social functioning* is measured through the Health of the Nation Outcome Scale (HoNOS), a scale that is standard in MHC in the UK [52]. The MHP scores each item on a scale from 0 to 4. The intra class correlation coefficient is 0.92, Cronbach’s alpha is 0.78 and it correlates well with other scales [53]. Moreover, the HoNOS is sensitive to measure change in people with SMI.

#### Additional and control measures

The following measures include instruments that are possible confounding, mediating or control variables.

1. *Socio-demographic characteristics* will be gathered at the start of the interview, including: age, gender, date of birth, number of children, marital status, nationality, education, living situation, income, and number of years in MHC.
2. *Current diagnosis* is gathered from the questionnaire for the MHP.
3. *Social Support* will be derived from the Inventory of Social Reliance (ISR) [54]. It consists of 11 items on emotional and practical support on a 4-point scale ranging from ‘almost never’ to ‘almost always’. The ISR is a frequently used questionnaire for people with SMI and has good psychometric properties [55].
4. *Neighborhood nuisance* is measured through Sections 1 and 2 of the Safety Monitor (see primary outcome measures for more information). These sections contain 9 questions about the experienced safety and contentment in and about their neighborhood.
5. *Perpetration* is assessed with the Safety Monitor. For the same criminal victimization incidents the participants are asked whether they were a perpetrator ever in their life, and if yes, also in the last year.
6. *FACT fidelity* is assessed through the fidelity scores from the CCAF, the Dutch organization that certifies F-ACT teams. The fidelity score is a mean score on 60 items that ranges from 0 to 5, where 0 means ‘no certificate’ and 5 means ‘optimal implementation’.
7. *Adherence to rehabilitation principles* is measured through a sample of the treatment plans of the clients that participate. This treatment plan consists of agreements, goals and wishes on several life domains for the following year.

### RCT analyses

#### Sample size

Sample size is calculated using the model of Twisk [56]. This model is suitable for multiple measurements over time, but can also be used for cluster randomization. With the ratio of the number of subjects in the compared groups being 1 (*r*), a correlation coefficient of the repeated measurements of 0.20 (ρ), a conservative difference between the groups in the mean value of social functioning of 0.25 (*v*), and a power of 1-β = 0.80, the number of participants needed is 151 for each condition at T2 measurement (α = 0.05, two-tailed). Taking into account an attrition rate of 15% for loss due to follow-up or consent withdrawal, 173 participants per condition need to be recruited to achieve the required power.

$$ N=\frac{Z\left(1-\alpha /2\right)+Z\left(1-\beta \right)\ 2\ \sigma 2\left(r+1\right)\left[1+\left(T-1\right)\rho \right]}{v2r\ T} $$

#### Statistical analyses

Data will be analyzed according to the ‘intention to treat’ principle, meaning that F-ACT teams (and thus clients) that are assigned to either experimental or control condition in randomization, will be analyzed accordingly. Because of the cluster design and multiple measurements over time, generalized linear mixed models (GLMM) will be used with SPSS (version 22); depending on the distribution of the outcome variable a logistic regression model or a linear regression model will be adopted. GLMM is robust with respect to missing data [57], which is not uncommon in research among outpatients with SMI. Therefore, multiple imputation is not necessary. To analyze the effect of the Victoria intervention on the main outcomes, differences in societal participation and victimization, both conditions will be compared after 9 and 18 months follow-up with time as a categorical variable. Measurements over time are nested within participants; therefore, random slopes will be added for time. Random intercepts will be added for the participants. The same procedure will be adopted for secondary outcome measures. Furthermore, possible confounders will be examined, such as socio-demographic characteristics, current diagnosis, number of years in MHC, or perpetration on whether they need to be added to the model. Only significant confounders will be added to the final model. In all analyses performed, two-tailed *p*-values < 0.05 are considered significant. Finally, Akaike’s Information Criterion (AIC) will be used for the selection of the final model that fits the data best and is the most generalizable [58].

## Discussion

Outpatients with SMI experience high rates of victimization, discrimination and stigmatization [11–17]. These adversities are important barriers to rehabilitation and societal participation [21]. This study is the first to evaluate an intervention aiming at recognition of victimization and other adversities, that also targets outpatients’ insight and coping skills with regard to possible risks to ensure safe societal participation. The aim of this first trial on the Victoria intervention is to gain insight into the implementation process and effectiveness of the Victoria intervention in reducing victimization (among other adversities) and increasing societal participation, on both the team level and the client level.

A major strength of this study will be the large sample size of outpatients with SMI, leading to sufficient power, which is often a problem in similar trials [59]. Only clients that are unable to fill out the questionnaire during the inclusion period of 6 months, due to insufficient understanding of the Dutch language, prolonged clinical admission, or severe symptomatology, will be excluded from the study. This, as well as the participation of multiple mental health centers, enhances generalizability of results.

This is, to our knowledge, the first study to address victimization and other adversities as a barrier for societal participation. In relation to this, we incorporated a broad range of outcome measures. Many previous studies on victimization only take into account clinical outcome measures or socio-demographic variables such as living situation [13, 14]. Although some studies include the influence of victimization on, for example, quality of life, this study includes a broad range of social outcome measures, such as social functioning and social support. These outcome measures are likely to be influenced by victimization or other adversities in the social domain [60, 61].

A final strength of the study is the multi-method approach. A process evaluation will be conducted to examine the implementation process of the intervention. Studies with a similar target population that examine complex interventions in a RCT often find no treatment effect, due to, for example, implementation or fidelity issues [62]. Within the design of an RCT, normally, there is little room for examining the implementation process, leading to a black box in explaining the results. Therefore, the UK Medical Research Council advises conducting a process evaluation, to understand context mechanisms and provide insights on implementation and fidelity [63]. For this reason, we do include a process evaluation and a longitudinal follow-up on effects, using a cluster RCT.

One of the main challenges in this study lies in the fact that the Victoria intervention is a complex intervention, encompassing characteristics of the local context, and the complexity of causal relations between intervention and outcomes [36]. MHPs in the participating teams sometimes work in multiple teams or, due to reorganization, they shift from one team to the other. This brings challenges in the implementation of the intervention. To address this, a strong collaboration is created between the two MHC institutions and the university in this project, with shared goals and input. Additionally, the process evaluation will be helpful in following the implementation process.

Another challenge of this study, and of most other studies among outpatients with SMI and follow-up measurements, is the dropout risk in the cluster RCT. In the sample size calculation, we take this into account by estimating a 15% loss to follow-up, and using effect sizes that may be considered conservative, compared to effect sizes found in other studies [64]. This leads to 347 required respondents at baseline measurement. Moreover, with the help of the MHPs, a contact plan, and by giving clients incentives for participating, we aim to prevent dropout as much as possible.

Finally, due to novelty of the Victoria intervention, there is no valid fidelity measure. Therefore, in this project a new fidelity checklist will be developed and used. To increase validity in measuring fidelity, we use triangulation by also including qualitative analyses of the recordings of the supervision meetings and questionnaires for both clients and MHP, as explained in the methods section.

In conclusion, the Victoria intervention is the first to incorporate a positive risk approach into a psychiatric rehabilitation method. This study is expected to provide scientific insights in ways to reduce victimization, (self-) stigmatization and discrimination, and increase societal participation, but also in the impact of other factors such as acknowledgement and awareness of these adversities. Moreover, results of this study may validate the Victoria intervention as one of the practices to better manage risk on adversities related to societal participation.

## Abbreviations

ACT: Assertive Community Treatment
AIC: Akaike’s Information Criterion
BPR: Boston University approach to Psychiatric Rehabilitation
CAU: Care As Usual
DISC-12: Discrimination and Stigmatization Scale
F-ACT: Flexible assertive community treatment
GLMM: Generalized Linear Mixed Models
HoNOS: Health of the Nation Outcome Scale
ISR: Inventory of Social Reliance
LQLP: Lancashire Quality of Life Profile
MANSA: Manchester Short Assessment of Quality of Life
MHC: Mental Health Care
MHCS: Mental Health Confidence Scale
MHP: Mental Health Professional
RCT: Randomized Controlled Trial
SFS: Birchwood Social Functioning Scale
SMI: Severe Mental Illness
TSQ: Trauma Screening Questionnaire
